# Validation of the Catatonia Quick Screen in the Original Bush‐Francis Catatonia Rating Scale Cohort

**DOI:** 10.1111/acps.70135

**Published:** 2026-08-14

**Authors:** James Luccarelli, Joshua Ryan Smith, Mark Kalinich, Jo Ellen Wilson, Jonathan P. Rogers, Stephan Heckers, Andrew Francis

**Affiliations:** ^1^ Harvard Medical School Boston Massachusetts USA; ^2^ Department of Psychiatry Massachusetts General Hospital Boston Massachusetts USA; ^3^ Department of Psychiatry and Behavioral Sciences Vanderbilt University Medical Center at Village of Vanderbilt Nashville Tennessee USA; ^4^ Vanderbilt Kennedy Center Vanderbilt University Nashville Tennessee USA; ^5^ Department of Psychiatry Beth Israel Deaconess Medical Center Boston Massachusetts USA; ^6^ Department of Veterans' Affairs Geriatric Research, Education and Clinical Center Tennessee Valley Healthcare System Nashville Tennessee USA; ^7^ Division of Psychiatry University College London London UK; ^8^ Department of Neuropsychiatry National Hospital for Neurology and Neurosurgery London UK; ^9^ Department of Psychiatry and Behavioral Sciences Pennsylvania State University Hershey Pennsylvania USA

**Keywords:** catatonia, diagnosis, psychiatric status rating scales, retrospective studies, screening

## Abstract

**Introduction:**

Delayed catatonia diagnosis may cause preventable harm. The 4‐item Catatonia Quick Screen (CQS) allows rapid screening, but its specificity requires evaluation. We assessed CQS sensitivity and specificity using the original Bush Francis Catatonia Rating Scale (BFCRS) cohort.

**Methods:**

Retrospective reanalysis of data from 215 consecutive inpatient psychiatric admissions. CQS classifications were compared against BFCRS, DSM‐5‐TR, and ICD‐11 criteria. 95% confidence intervals (CIs) were calculated via Clopper‐Pearson exact methods, treating the eight single‐sign patients conservatively as false positives.

**Results:**

All 15 catatonia cases screened positive (true positives). The CQS demonstrated 100% sensitivity (95% CI: 78.2% to 100%), 96.0% specificity (95% CI: 92.3% to 98.3%), 65.2% positive predictive value (95% CI: 42.7% to 83.6%), and 100% negative predictive value (95% CI: 98.1% to 100%).

**Conclusion:**

The CQS showed excellent sensitivity and high specificity in this psychiatric cohort. It serves as a rapid, low‐burden screening tool to accelerate clinical recognition, though positive results require formal diagnostic confirmation.

## Keypoints

- Significant Outcomes
  ○ The Catatonia Quick Screen (CQS) identified all catatonia cases in the original Bush‐Francis Catatonia Rating Scale cohort, supporting its sensitivity as a brief screening approach for catatonia in inpatient psychiatry.
  ○ Under conservative assumptions, the CQS demonstrated high specificity, suggesting that it may efficiently distinguish patients unlikely to have catatonia.
  ○ The findings support the CQS as an initial case‐identification tool.
- Limitations
  ○ The number of true positive catatonia cases was small, resulting in a wide confidence interval around the sensitivity estimate and limiting precision.
  ○ CQS status was derived from BFCSI/BFCRS item‐level ratings obtained during a broader catatonia assessment, rather than from an independently administered rapid four‐item assessment.
  ○ The analysis used a historical inpatient psychiatry cohort, which may limit generalizability to other clinical settings.

## 1 Introduction

Catatonia is a rare psychiatric disorder that affects patients of all ages and is characterized by changes in affect, cognition, and motor function [1]. Catatonia is associated with high morbidity and mortality, but is highly treatable with benzodiazepines and electroconvulsive therapy when promptly identified. Accordingly, delayed catatonia diagnosis can result in preventable patient harm.

While diagnostic criteria for catatonia have varied across time and diagnostic schema [2], the present *Diagnostic and Statistical Manual of Mental Disorders, Fifth Edition, Text Revision (DSM‐5‐TR)* requires at least three of 12 specified signs, while *the International Classification of Diseases, Eleventh Revision (ICD‐11)* requires 3 signs from a different list of 15 criteria. In clinical use, the Bush Francis Catatonia Rating Scale (BFCRS), a 23‐item rating scale generated in the inpatient psychiatry setting, is frequently used for catatonia assessment, with the first 14 of the items designated the Bush Francis Catatonia Screening Instrument (BFCSI) [3]. More recently, the Catatonia Quick Screen (CQS) [4], a 4‐item screening instrument, was developed to permit rapid, low‐burden identification of patients with possible catatonia. The CQS was generated from a multisite retrospective cohort of 446 pediatric and adult patients with catatonia who were assessed using the BFCSI, with item‐level data extracted for each of the 14 signs. Every possible combination of catatonic signs was then assessed as putative new screening instruments, prioritizing sensitivity while also considering clinical ease of assessment, applicability across catatonia presentations, and reliability of sign recognition.

Under the CQS, the presence of any one of four catatonic signs—excitement, mutism, staring, or posturing—demonstrated a sensitivity of 97% (95% CI: 95% to 98%) relative to the BFCSI and a 91% sensitivity (95% CI: 90% to 93%) in an independent validation sample of 1456 individuals. Although these findings established high sensitivity in real‐world clinical samples, the specificity of the CQS has not been rigorously evaluated.

Aims of the study: Here, we assess both the sensitivity and specificity of the CQS using the original inpatient cohort from which the BFCRS was derived.

## 2 Methods

This study is a reanalysis of data from the original BFCRS clinical cohort, which has been described previously [3]. Briefly, 215 consecutive admissions to an adult inpatient psychiatry ward were assessed for catatonia using the BFCSI. Of these, 15 had two or more signs, eight had one sign, and 192 had no signs. The specific catatonic signs were recorded for the 15 cases with two or more but were not recorded for the eight patients with a single feature.

In this reanalysis, the sensitivity and specificity of the CQS were assessed by determining whether each individual would screen positive for catatonia using the CQS and comparing this classification with BFCRS, DSM‐5‐TR, and ICD‐11 criteria. To provide conservative estimates, 95% confidence intervals were calculated using Clopper–Pearson exact methods. As this is a reanalysis of previously‐published, de‐identified data, it qualifies as not human subjects research.

## 3 Results

Among the 215 patients, 192 had no BFCRS features and therefore would screen negative on the CQS and not meet criteria for catatonia under BFCRS, DSM‐5‐TR, or ICD‐11 (true negatives). Eight patients had a single catatonic sign and do not meet criteria for catatonia under BFCRS, DSM‐5‐TR, or ICD‐11. To conservatively estimate CQS specificity, these eight cases were treated as if their single unrecorded sign was included on the CQS (false positives). Among the 15 patients with two or more catatonic signs, all would score positive using CQS and meet criteria for catatonia under BFCRS, DSM‐5‐TR, and ICD‐11 (true positives) (Table 1). No cases of catatonia would have been missed by the CQS (false negatives = 0). As a result, in this cohort the CQS demonstrated a sensitivity of 100% (95% CI: 78.2% to 100%), a worst‐case specificity of 96.0% (95% CI: 92.3% to 98.3%), a positive predictive value of 65.2% (95% CI: 42.7% to 83.6%), and a negative predictive value of 100% (95% CI: 98.1% to 100%).

**TABLE 1:** Individual catatonic signs of the 15 patients with catatonia in the overall 215 patient cohort.

Patient	1	2	3	4	5	6	7	8	9	10	11	12	13	14	15
1. Excitement	**1**									**1**					**1**
2. Immobility/stupor:	1	1	1	1	1	1	1	1	1		1	1	1	1	
3. Mutism:		**1**	**1**	**1**	**1**	**1**	**1**	**1**	**1**		**1**	**1**	**1**	**1**	**1**
4. Staring:		**1**	**1**		**1**	**1**	**1**	**1**	**1**		**1**	**1**	**1**	**1**	
5. Posturing/catalepsy:		**1**	**1**	**1**				**1**	**1**	**1**	**1**	**1**	**1**	**1**	**1**
6. Grimacing:			1		1					1		1	1	1	1
7. Echopraxia/echolalia:										1				1	1
8. Stereotypy:						1									1
9. Mannerisms:	1				1										1
10. Verbigeration:									1	1					1
11. Rigidity:							1	1			1	1	1	1	
12. Negativism:				1	1	1	1		1	1	1	1	1	1	1
13. Waxy flexibility:									1						
14. Withdrawal:		1		1		1	1	1		1	1	1	1	1	
BFCSI Score	3	5	5	5	6	6	6	6	7	7	7	8	8	9	9
Meets DSM‐5‐TR Criteria?	Yes	Yes	Yes	Yes	Yes	Yes	Yes	Yes	Yes	Yes	Yes	Yes	Yes	Yes	Yes
Meets ICD‐11 criteria?	Yes	Yes	Yes	Yes	Yes	Yes	Yes	Yes	Yes	Yes	Yes	Yes	Yes	Yes	Yes
CQS positive?	Yes	Yes	Yes	Yes	Yes	Yes	Yes	Yes	Yes	Yes	Yes	Yes	Yes	Yes	Yes

*Note:* CQS items are bolded.

## 4 Discussion

In a reanalysis of the original inpatient psychiatric cohort from which the BFCRS and BFCSI were derived, the CQS demonstrated excellent sensitivity (100%) and high specificity (96%) in identifying cases of catatonia. This performance is comparable to the 97% sensitivity of the CQS in the original multi‐site inpatient medical cohort from which it was derived [4]. The small number of catatonia cases in this cohort (*n* = 15) results in a relatively wide confidence interval around the sensitivity estimate, which should be interpreted with caution and may reflect imprecision inherent to the limited sample size. Importantly, the specificity estimate reflects a conservative analytic assumption, treating all eight patients with a single unspecified catatonic sign as false positives. Accordingly, the observed specificity likely represents a lower bound. This supports the use of the CQS as a rapid screener for catatonia in the inpatient psychiatric setting.

The CQS possesses several characteristics that support its clinical utility. All four CQS items rely exclusively on observable behaviors and do not require a physical examination, substantially lowering the threshold for assessment compared with more comprehensive instruments such as the 14‐item BFCSI. Each CQS item showed moderate to high recognition rates in a standardized video‐based assessment [5], suggesting that the included signs are readily identifiable in clinical practice. In addition, the CQS items use non‐esoteric terminology and intentionally sample both hyperactive and hypoactive manifestations of catatonia, addressing a common source of under‐recognition. The brevity of the CQS—typically requiring less than 60 s to complete—and its feasibility for administration via telemedicine or from across the room further enhance its practicality. Taken together, these features suggest that the CQS may be particularly well suited for initial case identification in busy inpatient and outpatient environments, where more detailed assessments may be impractical or delayed.

As the positive predictive value of 65.2% illustrates, however, the CQS is not intended to replace comprehensive diagnostic assessment, and positive screens should prompt confirmatory assessment using established catatonia rating scales. Moreover, the validity of the CQS for identifying catatonia in settings beyond inpatient psychiatry, where the BFCRS was originally derived, is an important area of future research. Additionally, because CQS ratings in this reanalysis were derived from BFCSI/BFCRS item‐level data obtained during a broader catatonia assessment, rather than from an independently administered rapid CQS assessment, an area for future prospective studies should be to validate CQS performance against a reference‐standard assessment using independent raters.

## 5 Conclusion

In the original inpatient cohort of 215 consecutive psychiatric admissions used to derive the BFCRS, the CQS demonstrates excellent sensitivity (100%) and high specificity (96%) in identifying cases of catatonia while requiring the assessment of only four observable signs. These findings further support the CQS as a rapid screening instrument for catatonia that may facilitate earlier recognition of this highly treatable neuropsychiatric disorder. Further work is required to validate the CQS in other care environments and as a prospective screening tool.

## Author Contributions

J.L.: conceptualization, methodology, formal analysis, writing – original draft, writing – review and editing; J.R.S.: investigation, writing – review and editing. M.K.: formal analysis, writing – review and editing; J.E.W.: investigation, resources, writing – review and editing; S.H.: investigation, writing – review and editing; J.P.R.: validation, writing – review and editing; A.F.: data curation, validation, writing – review and editing.

## Funding

This work was supported by the National Institute for Health and Care Research, UK (CL‐2023‐18‐002; JPR). The sponsors had no role in study design, writing of the report, or data collection, analysis, or interpretation.

## Conflicts of Interest

J.L. receives funding from Harvard Medical School Dupont Warren Fellowship and Livingston Awards, the Rappaport Foundation, the American Academy of Child and Adolescent Psychiatry, and the Foundation for Prader‐Willi Research. He has received equity and consulting fees from Revival Therapeutics Inc. and consulting fees from Soleno Therapeutics. J.R.S. receives funding from the Eunice Kennedy Shriver National Institute of Child Health and Human Development, National Institute of Mental Health, Axial Therapeutics, Janssen Pharmaceuticals, Vanda Pharmaceuticals, Bristol Myers Squibb, and Roche. M.K. has received compensation from Watershed Informatics and has equity in Watershed Informatics, Revival Therapeutics, nference, Robot on Rails, and OpenEvidence. J.E.W. receives support from the Department of Veterans Affairs, Geriatric, Research, Education and Clinical Center (GRECC) at the Tennessee Valley Healthcare System in Nashville, TN. Additionally, J.E.W. receives funding from NIH, Ono Pharmaceutical, AC‐Immune, and Bristol Myers Squibb J.P.R. reports research funding from Wellcome and NIHR; royalties from Taylor & Francis; payment for reviewing from Johns Hopkins University Press; and speaker fees from the Alberta Psychiatric Association, Grey Nuns Hospital (Edmonton), Informed Research & Training Ltd., North East London NHS Foundation Trust, TooFar Media, Vanderbilt University Medical Center, and the Committee for Biological Psychiatry in Norway. J.P.R. has served as a member of an advisory board for Teva. J.P.R. has received support to attend meetings from the British Association for Psychopharmacology, the European Congress of Neuropsychopharmacology, and the Royal College of Psychiatrists. J.P.R. is a Council member for the British Association for Psychopharmacology, a member of the Medical Advisory Board of the Catatonia Foundation and an Advisor to the Global Neuropsychiatry Group. J.P.R. conducts expert witness work. S.H. has received funding from NIH. All other authors declare no conflicts of interest.

## Data Availability

All de‐identified data used in this analysis is presented in the manuscript.
